# A Comprehensive Review of Deep Learning Applications in Cotton Industry: From Field Monitoring to Smart Processing

**DOI:** 10.3390/plants14101481

**Published:** 2025-05-15

**Authors:** Zhi-Yu Yang, Wan-Ke Xia, Hao-Qi Chu, Wen-Hao Su, Rui-Feng Wang, Haihua Wang

**Affiliations:** 1 College of Information and Electrical Engineering, China Agricultural University, 17 Qinghua East Road, Haidian, Beijing 100083, China; yangzhiyu@cau.edu.cn (Z.-Y.Y.); 2020301010225@cau.edu.cn (W.-K.X.); 2 College of Land Science and Technology, China Agricultural University, 17 Qinghua East Road, Haidian, Beijing 100083, China; chuhaoqi@cau.edu.cn; 3 College of Engineering, China Agricultural University, 17 Qinghua East Road, Haidian, Beijing 100083, China; 4 National Innovation Center for Digital Fishery, China Agricultural University, Beijing 100083, China

**Keywords:** cotton, cotton cultivation, cotton growth management, cotton harvesting and processing, deep learning, precision agriculture

## Abstract

Cotton is a vital economic crop in global agriculture and the textile industry, contributing significantly to food security, industrial competitiveness, and sustainable development. Traditional technologies such as spectral imaging and machine learning improved cotton cultivation and processing, yet their performance often falls short in complex agricultural environments. Deep learning (DL), with its superior capabilities in data analysis, pattern recognition, and autonomous decision-making, offers transformative potential across the cotton value chain. This review highlights DL applications in seed quality assessment, pest and disease detection, intelligent irrigation, autonomous harvesting, and fiber classification et al. DL enhances accuracy, efficiency, and adaptability, promoting the modernization of cotton production and precision agriculture. However, challenges remain, including limited model generalization, high computational demands, environmental adaptability issues, and costly data annotation. Future research should prioritize lightweight, robust models, standardized multi-source datasets, and real-time performance optimization. Integrating multi-modal data—such as remote sensing, weather, and soil information—can further boost decision-making. Addressing these challenges will enable DL to play a central role in driving intelligent, automated, and sustainable transformation in the cotton industry.

## 1. Introduction

**Figure 1:**
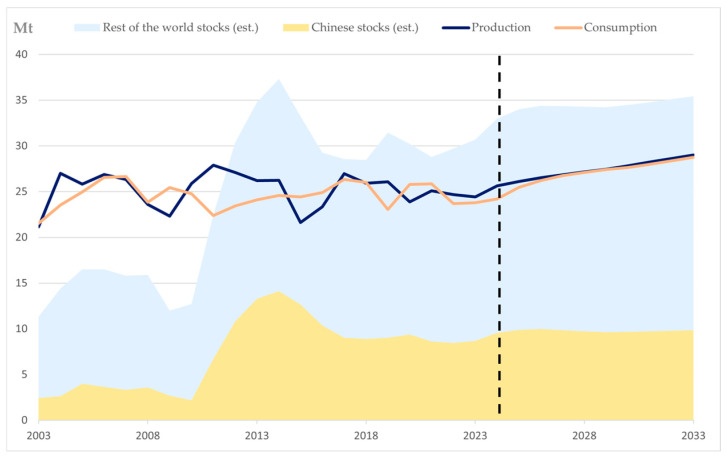
OECD-FAO predictions of future cotton production and consumption based on past data. Note: est. stands for estimate. Source: OECD/FAO (2024), “OECD-FAO Agricultural Outlook” OECD Agriculture statistics (database), https://www.fao.org/markets-and-trade/food-and-agricultural-markets-analysis-FAMA/cotton/1/en (accessed on 4 April 2025).

Cotton is one of the most important economic crops globally, playing a central role in both agricultural production and the textile industry [1]. From an agricultural perspective, cotton cultivation is widely distributed across multiple countries and regions, serving as a primary economic source for numerous farmers [2,3,4,5,6]. Statistical data indicate that the global cotton planting area remained relatively stable over the years, while fluctuations in its yield directly impact the supply and demand dynamics of the international agricultural market. In addition, the OECD-FAO Agricultural Outlook shows that global production of cotton is expected to grow steadily and reach 29 Mt by 2033, 17% higher than in the base period, which indicates that the demand for cotton is increasing year by year (Figure 1). In the textile industry, cotton is the primary source of natural fiber, valued for its softness, breathability, and moisture absorption properties, making it widely used in clothing, home textiles, and other applications [7,8]. From everyday apparel such as T-shirts and jeans to household items such as bedding and curtains, the extensive utilization of cotton products allowed it to maintain a dominant position in the textile raw material market. While meeting essential human needs, cotton also continues to drive the sustainable development of the textile industry [9,10].

Despite advancements, traditional cotton cultivation and processing still face significant challenges [11,12,13,14,15,16]. During cultivation, frequent pest infestations—such as bollworms and red spiders—and diseases such as Fusarium wilt and Verticillium wilt pose serious threats to yield and fiber quality. Farmers often rely heavily on chemical pesticides, which increases production costs, contributes to environmental pollution, and raises concerns over pesticide residues and food safety. Cotton production is also highly water-intensive; in water-scarce regions, irrigation limitations constrain planting scale and yield potential. On the processing side, many operations—such as post-harvest cleaning, ginning, spinning, and weaving—remain largely manual and lack automation. This results in low efficiency, extended processing times, high costs, and inconsistent product quality, making it difficult for the traditional cotton industry to meet the modern textile sector’s demand for high-quality, standardized cotton products.

In recent years, the rapid advancement of artificial intelligence spurred the growing application of deep learning (DL) in agriculture, demonstrating considerable potential across various domains [17,18,19,20,21,22]. By constructing complex neural network models, deep learning enables efficient analysis and processing of large-scale data, facilitating precise predictions and intelligent decision-making [23,24]. In agricultural production, it has been widely applied to crop growth monitoring, pest and disease forecasting, and soil fertility assessment [24,25]. For the cotton industry, the adoption of deep learning technologies holds promise for transformative improvements.

During the cultivation stage, deep learning algorithms can be employed to analyze remote sensing images, meteorological data, and soil information throughout the cotton growth cycle, enabling accurate predictions of pest and disease outbreaks in terms of timing and spatial distribution. This facilitates intelligent early warning and targeted control, thereby reducing pesticide usage and enhancing the precision of disease management. Additionally, deep learning can optimize irrigation and fertilization strategies based on the specific needs of different growth stages, improving the efficiency of water and nutrient utilization while minimizing resource waste. In the processing stage, deep learning can be integrated into the intelligent control of automated equipment, enabling precise monitoring and optimization of processing workflows. This not only enhances production efficiency and product quality, but also lowers operational costs, promoting the transition of the cotton industry toward greater intelligence and efficiency.

Despite its promising prospects in agriculture, the practical application of deep learning in the cotton industry still faces several challenges and limitations. First, training deep learning models requires large volumes of high-quality data; however, the acquisition of agricultural data is often constrained by environmental conditions, equipment capabilities, and technical expertise, potentially resulting in insufficient data quality and quantity, which hampers model performance. Second, the high computational complexity of deep learning models demands substantial computing resources, which may limit their applicability in resource-constrained agricultural settings. Furthermore, the implementation of deep learning technologies depends on supporting infrastructure and technical expertise, which may be lacking in remote or economically underdeveloped regions, thus hindering widespread adoption and dissemination.

**Figure 2:**
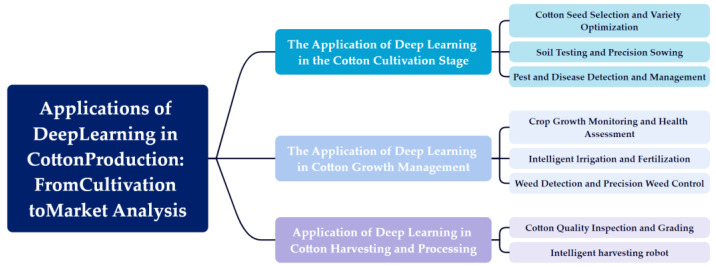
The application of deep learning in various processes of cotton production.

As shown in Figure 2, to systematically explore the application of deep learning technologies in the cotton industry, this paper provides a review in the following areas:

1. The application of deep learning in the cotton cultivation stage;
2. The application of deep learning in cotton growth management;
3. Application of deep learning in cotton harvesting and processing.

The structure of this paper is as follows: Section 2 reviews the application of deep learning technologies in the cotton planting stage; Section 3 elaborates on the use of deep learning in cotton growth management; Section 4 provides an overview of deep learning applications in cotton harvesting and processing; Section 5 discusses the current challenges and future directions in the cotton industry chain; and Section 6 concludes with a summary and outlook.

To enhance the transparency and reproducibility of the literature review, this study adhered to the preferred reporting items for systematic reviews and meta-analyses (PRISMA 2020) guidelines for systematic reviews. A comprehensive screening and analysis of the relevant literature were conducted.

The literature search was performed on Google Scholar, with a time range from January 2010 to December 2024, encompassing both English and Chinese sources. The following keywords were used in combination for the search: “cotton”, “deep learning”, “machine vision”, “precision agriculture”, and “image recognition”, with Boolean logic expressions (AND/OR) applied for flexible combinations.

To ensure the selected literature was highly specialized and relevant, a set of criteria was established, as outlined in Table 1.

**Figure 3:**
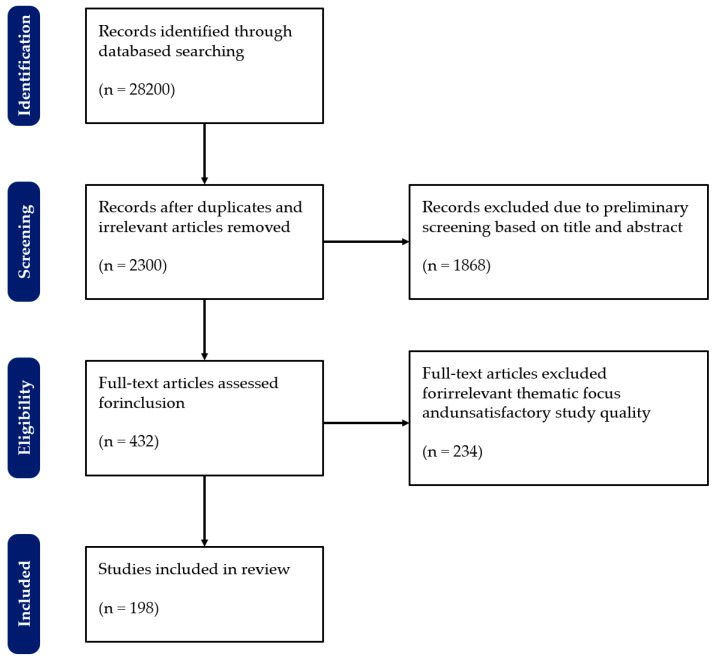
Literature screening flowchart.

Based on the above selection and analysis criteria, we conducted a literature search. The literature screening process is illustrated in Figure 3.

To ensure the methodological rigor of the included studies, we conducted a qualitative quality assessment. The evaluation criteria included dataset size, model validation methods, the presence of field testing, and the completeness of performance metrics. Studies that utilized large-scale or publicly available datasets, performed cross-validation or independent testing, and were tested in real-world environments were considered more methodologically rigorous. In contrast, some studies had limitations in validation procedures or application scenario testing, which may affect the generalizability of their results. Although this study did not employ a formal scoring scale, the aforementioned factors were thoroughly considered in the analysis and discussion.

Considering the task differences between models in the study, the evaluation metrics used were not entirely identical. We classified the different tasks performed by deep learning models in agriculture and provided the indicated metric data as accurately as possible for each task. The classification and evaluation metrics are shown in Table 2.

## 2. The Application of Deep Learning in the Cotton Cultivation Stage

### 2.1. Cotton Seed Selection and Variety Optimization

#### 2.1.1. Deep Learning-Based Cotton Seed Quality Detection

Genetic molecular marker technology enables high-precision identification of seed varieties, but it is costly and requires specialized expertise [26]. The advancement of deep learning offers an efficient, non-destructive, and automated solution for intelligent seed screening [27], with strong capabilities in handling high-dimensional data and pattern recognition. This enhances the accuracy and efficiency of seed selection and provides a solid foundation for the development of precision agriculture.

Variety identification is central to seed screening, as the purity of cotton seeds directly influences both yield and quality, making rapid and accurate classification essential. Zhu et al. [28] employed near-infrared hyperspectral imaging combined with a custom-designed CNN model to classify seven cotton seed varieties from Xinjiang, achieving an accuracy exceeding 80%. Liu et al. [29] integrated hyperspectral and texture features, using an extreme learning machine model to classify five cotton varieties, with training and testing accuracies of 100% and 98.89%, respectively. Du et al. [30] embedded the CBAM attention mechanism into a ResNet50 network and conducted experiments on a large-scale dataset, achieving an identification accuracy of 97.23% with an image processing time of only 0.11 s per image, demonstrating high efficiency. In addition, seed vigor, which affects germination rate and final yield, is another critical trait. Li et al. [31] proposed a non-destructive vigor detection method based on a one-dimensional CNN combined with a gray-level co-occurrence matrix, attaining a correlation coefficient (R) of 0.9427 and a RMSE of 0.6872. This approach outperformed single-modality detection, confirming the effectiveness of deep learning in seed vigor assessment.

**Figure 4:**
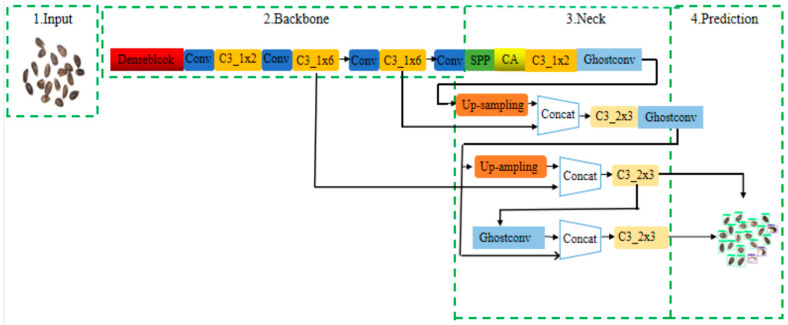
Improved YOLOv5s model diagram [33].

Seed quality plays a crucial role in determining cotton fiber quality, making efficient evaluation highly significant. Liu et al. [32] enhanced the YOLOv5 model for cotton seed damage detection by replacing the Focus module with DenseBlock, introducing a coordinate attention mechanism, substituting standard convolutional layers with GhostConv, simplifying the detection head, and incorporating the CIOU loss function for bounding box regression. As illustrated in Figure 4, their model achieved a mAP@50 of 99.5% and a recall rate of 99.3% for uncoated seeds, and 99.2% and 98.9%, respectively, for coated seeds—demonstrating improved accuracy with reduced computational complexity. Liu et al. [33] further proposed a lightweight variant of YOLOv5s, achieving a detection accuracy of 92.4%, recall of 91.7%, and mAP of 98.1%, with an inference speed of 97 fps, enhancing both precision and efficiency. Zhang et al. [34] developed a dual-camera system and introduced the Light-YOLO model, achieving an online detection accuracy of 86.7%, thereby validating the feasibility of real-time detection.

In summary, deep learning achieved significant progress in cotton seed variety identification, vigor prediction, and damage detection. By integrating hyperspectral imaging, texture analysis, and improved neural network models, variety classification accuracies exceeded 98%. Enhanced YOLO-based models improved detection precision and computational efficiency, enabling accurate and efficient seed quality assessment. Despite the promising performance of deep learning-based seed quality detection methods across various experimental datasets, several challenges remain in practical applications:

- High equipment costs and deployment constraints: hyperspectral imaging systems and deep learning models require substantial computational resources, which may limit their adoption in resource-constrained agricultural settings.
- Data acquisition difficulties: The training of deep learning models depends on large-scale, high-quality datasets. However, agricultural data collection is often constrained by environmental conditions, equipment limitations, and technical capacity, potentially leading to insufficient data quality and quantity, which undermines model performance.
- Limited model generalization: The variability of agricultural environments—including lighting conditions, soil types, and climatic changes—can affect model generalization. More robust models are needed to address these challenges.

Looking ahead, with advances in computer vision, improvements in data acquisition technologies, and the development of lightweight deep learning models, cotton seed quality detection based on deep learning is expected to be more widely applied in agricultural production, providing strong technical support for precision agriculture.

#### 2.1.2. Cotton Genomic Data Analysis and Variety Improvement

Genome-wide identification and expression analysis are of significant value in deciphering key cotton genes, revealing genetic mechanisms, and guiding precision breeding [35,36]. These methods help identify critical genes regulating cotton yield, stress resistance, and fiber quality, providing a theoretical basis for variety improvement [37,38]. Due to the limited inclusion of cotton genes in plant databases, researchers recently developed dedicated cotton genome databases [39,40,41,42], laying the data foundation for the application of deep learning in gene analysis, trait prediction, and breeding optimization.

The application of deep learning advanced the functional analysis of cotton genes and the prediction of complex traits. Li et al. [43] combined high-throughput phenotypic analysis with GWAS to investigate the genetic variation of cotton fruit branch angles, providing genetic references for plant type optimization. Zhang et al. [44] developed an integrated model combining LightGBM, XGBoost, and random forest, based on 121 biochemical traits, to improve the prediction accuracy of cotton cold stress resistance genes, achieving an accuracy of 80.80%. Zhao et al. [45] integrated eQTL analysis with XGBoost to explore the genetic regulatory network of cotton seed yield, identifying key genes such as NF-YB3, FLA2, and GRDP1, and revealing their genetic regulatory mechanisms.

Cotton variety optimization is crucial for yield, stress resistance, and fiber quality [46]. Li et al. [47] employed the U-Net-3D model combined with a threshold segmentation algorithm to perform morphological reconstruction on 102 cotton germplasm resources, extracting 11 basic phenotypic traits and 3 new indices (seed coat specific surface area, seed coat thickness ratio, and seed density ratio), thereby improving the efficiency of variety optimization.

This section reviews the application of genomic data analysis in cotton breeding, emphasizing the roles of genome-wide identification, expression analysis, and database construction. Research shows that the integration of deep learning and machine learning can help elucidate gene functions, predict important traits, and optimize breeding strategies. Additionally, deep learning-based phenotypic reconstruction and feature extraction of germplasm resources provide new technological approaches for precision breeding.

Despite significant progress in this field, the following challenges remain:

- High costs and computational resource limitations: Genome-wide identification and expression analysis are costly, which may limit their large-scale application in resource-limited agricultural environments. Moreover, the computational resource requirements of deep learning models pose a challenge for research teams or agricultural enterprises with limited hardware capabilities.
- Database compatibility issues: although cotton-specific genomic databases have been gradually improved in recent years, poor compatibility between different databases makes data integration and sharing difficult, thus hindering the efficient use of data.
- Data annotation and model generalization: The application of deep learning in genomic research relies heavily on high-quality annotated data. However, data acquisition in agriculture is constrained by environmental conditions and experimental design, leading to varying data quality. Furthermore, the complexity of genomic data means that the generalization ability of deep learning models needs further optimization to enhance their adaptability to different environments and breeding contexts.

In the future, with the advancement in high-throughput sequencing technologies, the optimization of deep learning algorithms, and improvements in data-sharing mechanisms, the application of genomic data analysis in cotton variety improvement will have broader prospects. By integrating multi-omics data, developing deep learning models with stronger generalization capabilities, and enhancing the standardization of databases, the efficiency and reliability of precision breeding can be further improved.

### 2.2. Soil Testing and Precision Sowing

#### 2.2.1. Application of Remote Sensing and Computer Vision in Soil Quality Assessment

Soil quality has a direct impact on cotton growth and yield, making accurate monitoring and scientific assessment essential. Key indicators include nitrogen fertilizer application, duration of no-tillage, soil organic carbon, and the use of cover crops [48]. Traditional agriculture often relies on experience-based management, which can lead to imprecise fertilization and irrigation, resulting in soil degradation, resource waste, and yield decline. With the advancement of remote sensing technologies, large-scale soil data can now be acquired through satellite imagery, UAV-based imaging, and ground-based spectral measurements, enabling real-time and precise soil quality assessment.

In recent years, numerous studies integrated remote sensing with field sampling to investigate the influence of soil properties on cotton yield. Aghayev et al. [49] assessed soil productivity in a 15-hectare cotton field using a combination of remote sensing and laboratory analysis. Jia et al. [50] proposed a low-cost, high-precision nitrogen management approach by integrating UAV-based remote sensing, optimizing fertilization strategies under drought conditions and improving nitrogen use efficiency. Carneiro et al. [51] applied machine learning and remote sensing data to analyze variables such as soil parameters and topographic indices, identifying soil electrical conductivity, Sentinel-2 spectral data, topographic position index (TPI), LiDAR, and RTK elevation data as key predictors of cotton yield.

In addition, Qi et al. [52] combined satellite, UAV, and ground-based spectral monitoring to assess soil salinization in coastal cotton-growing areas and proposed a cost-effective and efficient approach for soil information acquisition. Tian et al. [53] integrated UAV imagery with deep learning to evaluate the effect of planting density on cotton emergence uniformity, finding that sandy soils with low electrical conductivity (ECa) favored uniform emergence, whereas clay soils with high ECa reduced emergence consistency. Feng et al. [54] developed a deep learning method based on multi-source data fusion to quantify the impact of soil texture and weather conditions on cotton yield, achieving an error range of 8.9% to 13.7%, thereby confirming the method’s effectiveness.

**Figure 5:**
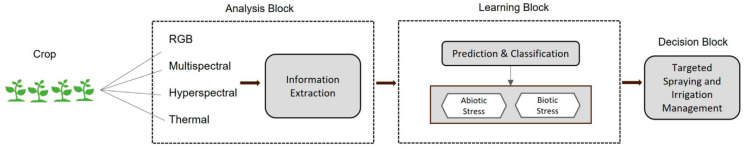
Vision guided stress detection in crops [55].

Computer vision and deep learning have also been widely applied to soil monitoring. Ghazal et al. [55] developed a vision-guided soil monitoring system that integrates image processing and machine learning to predict soil properties. They proposed an automated vision-guided framework for detecting biotic and abiotic stresses in soil, with the system workflow illustrated in Figure 5. Zhao et al. [56] introduced a multi-layer spectral calibration strategy and employed methods such as Cubist and bagging-PLSR to predict the physicochemical properties of different soil layers, demonstrating the feasibility of spectral calibration in soil monitoring.

This section reviews the applications of remote sensing and computer vision techniques in soil quality assessment for cotton fields, highlighting their critical role in precision agriculture. Studies have shown the following:

- When combined with field sampling, laboratory analysis, and machine learning, remote sensing effectively monitors key soil parameters such as organic carbon, nitrogen content, and salinization, optimizing management strategies.
- UAV imagery, satellite data, and ground-based spectral measurements support nitrogen management, planting density optimization, yield prediction, and soil degradation monitoring.
- Deep learning demonstrated superior performance in soil quality assessment and prediction, enabling efficient and accurate analysis of soil data.

Despite significant progress in this field, several challenges remain:

- Limited spatial resolution hampers detection of fine-scale soil variations, constraining precision in localized management.
- Multi-source data fusion is hindered by inconsistencies in format, resolution, and quality across datasets, complicating integration and error correction.
- Environmental variability—including climate change, pest outbreaks, and anthropogenic factors—affects soil quality, yet current models insufficiently account for such dynamic influences.

Looking ahead, advancements in remote sensing technology, improvements in computer vision and deep learning algorithms, and the refinement of multi-source data fusion methods are expected to further enhance the accuracy and automation of soil quality assessment. To address the aforementioned challenges, future research may focus on the following directions:

- Acquisition and application of high-resolution remote sensing data to improve the detection of small-scale variations in soil properties;
- Optimization of multi-source data fusion techniques to increase compatibility across data sources and improve the efficiency of information integration;
- Developing robust deep learning models adaptable to environmental variability, improving the reliability of soil quality assessments in precision agriculture.

#### 2.2.2. Deep Learning-Based Intelligent Sowing System

Intelligent seeding systems are a critical component of precision agriculture, aiming to improve seeding efficiency, reduce seed waste, and optimize planting density to enhance crop yield and resource utilization [57]. Traditional seeding methods primarily rely on mechanical adjustments [58] and lack capabilities for real-time monitoring and dynamic regulation, often resulting in issues such as uneven seeding, missed spots, or overseeding [59,60]. With the advancement of deep learning, computer vision, and Internet of Things (IoT) technologies, intelligent seeding systems rapidly developed in the fields of agricultural automation and precision seeding, offering more accurate and efficient solutions for modern farming. These systems leverage artificial intelligence, big data analytics, and smart sensors to monitor the seeding process in real time, optimize seeding parameters, and enhance the overall level of agricultural intelligence [61].

In recent years, deep learning and computer vision technologies have been increasingly applied in intelligent seeding systems. For instance, Bai et al. [62] developed a seeding quality monitoring system for precision hill-drop cotton planters based on automatic color matching detection and visual analysis. The system can record seeding quantity, missed seed events, planter forward speed, and operational area in real time, achieving a seeding monitoring accuracy of 93% and a missed seeding detection accuracy of over 91%. The application of these technologies significantly improved the accuracy and stability of cotton seeding while reducing resource waste during the seeding process.

Crop emergence rate is one of the key agronomic parameters in field management, typically evaluated based on plant count, spacing uniformity, and seedling growth status [63]. In recent years, several studies focused on utilizing intelligent seeding systems to automatically estimate the number of newly emerged cotton plants, thereby assisting growers in making informed decisions about the need for replanting [64,65]. For example, Xu et al. [66] developed an efficient cotton seedling detection and assessment system by integrating remote sensing index calculation, threshold segmentation, morphological feature analysis, and a line-based method for weed removal. This system enables comprehensive evaluation of field growth conditions and provides valuable guidance for precision agricultural management.

This section reviews the research progress of intelligent seeding systems in precision agriculture, highlighting the vital role of deep learning and computer vision in improving seeding accuracy and emergence assessment. Studies demonstrated that:

- Vision- and deep learning-based intelligent systems can monitor seeding quality in real time, optimize parameters, and promote agricultural automation.
- Combining UAV remote sensing, morphological analysis, and intelligent algorithms improves cotton emergence detection and supports replanting decisions.

Despite the significant progress of intelligent seeding systems, several challenges remain:

- Environmental adaptability: current systems need better resilience to varying soil types, climates, and crop species.
- Multimodal fusion and precision decision-making: integrating remote sensing, ground monitoring, and DL to refine seeding strategies is still under development.
- High hardware costs: dependence on precision sensors and automated machinery limits adoption, especially in resource-limited regions.

Ongoing advancements in computer vision, remote sensing, and smart equipment will likely enhance planting precision and overall productivity. Future research should focus on the following:

- Enhancing model adaptability to improve the generalization of intelligent seeding systems under diverse field conditions;
- Optimizing data fusion strategies by integrating multi-source data—including remote sensing, ground-based monitoring, and machinery operation data—to improve seeding precision;
- Reducing hardware costs and increasing system accessibility to promote the widespread adoption of intelligent seeding systems in global agricultural production.

### 2.3. Pest and Disease Detection and Management

#### 2.3.1. Diagnosis of Cotton Pests and Diseases

Cotton is highly susceptible to bacterial, fungal, and viral diseases, while traditional manual inspection methods are inefficient and prone to misdiagnosis in large-scale cultivation settings [25,67]. Deep learning—particularly computer vision techniques—can significantly enhance the accuracy and efficiency of cotton disease detection [68,69,70].

In recent years, image classification models achieved notable breakthroughs in cotton disease detection. Zekiwos et al. [71] developed a CNN model capable of identifying angular leaf spot, leaf blight, and red spider mite infestations with an accuracy of 96.4%. Memon et al. [72] combined CNN, VGG16, ResNet50, and meta-learning to construct a dataset and optimize the model, achieving a detection accuracy of 98.53%. Ganguly et al. [73] employed a hybrid architecture of DenseNet121 and ResNet50 to accurately classify six types of cotton diseases, reaching a classification accuracy of 96%. Remya et al. [74] integrated a Vision Transformer with acoustic sensors and trained the model on 32,000 cotton disease images, achieving an accuracy of 99%, outperforming MobileNet, ResNet152v2, and VGG16. Lin et al. [75] enhanced ResNet by incorporating the CBAM attention mechanism and multi-scale feature fusion, resulting in a 5.416% improvement in recognition accuracy and a 1.110% reduction in false positive rate.

Object detection algorithms also made significant advances in cotton disease and pest identification. Zhang et al. [76] enhanced YOLOX to effectively detect five types of diseases, achieving a mAP of 94.60%, outperforming YOLOv5 and Faster R-CNN. Similarly, Li et al. [77] optimized YOLOv8s, reaching a mAP of 93.7% with a reduced detection time of 8.01 ms. In addition, Gao et al. [78] integrated Transformer, knowledge graph, and edge computing, resulting in an accuracy of 94%, a mAP increase to 95%, and a frame rate (FPS) of 49.7. Yang et al. [79] proposed SRNet-YOLO, which combines super-resolution techniques with the BiFormer attention mechanism to enhance the detection of tiny pests.

Additionally, Rafael Faria Caldeira et al. [80] conducted cotton leaf image collection at a commercial cotton plantation in Unaí, Minas Gerais, Brazil, and utilized GoogleNet and ResNet50 networks for cotton leaf lesion recognition, achieving accuracy rates of 86.6% and 89.2%, respectively, which were nearly 10% higher than traditional machine learning methods such as SVM and KNN. Pakistani scholar Nimra Pechuho et al. [81] retrained the Inception-V3 model for cotton disease detection, ultimately achieving a test accuracy of 91%.

To enhance the quantitative aspect of the review, we provide a comparative summary of the model performance metrics in this section. In cotton-related tasks, the accuracy of deep learning models typically ranges from 85% to 99%, with F1 scores generally exceeding 0.90, and mAP values for object detection tasks often surpassing 90%. In contrast, traditional machine learning methods (such as SVM, random forests, or ELM) generally achieve accuracy below 80%, with limited generalization ability in multi-class or complex tasks. This trend indicates that, under conditions with large and diverse datasets, deep learning models generally outperform traditional models in classification and detection tasks.

**Figure 6:**
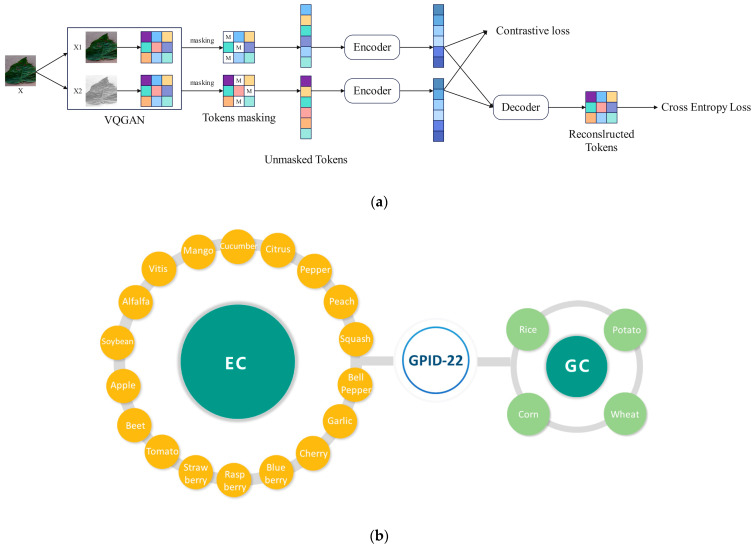
(**a**) Structural diagram of CRE framework; (**b**) taxonomy of the GPID-22 dataset [25].

It is worth noting that in our previous study [25], we proposed a self-supervised pretraining method for plant disease and pest classification based on the Vision Transformer, named CRE, and constructed a large-scale dataset (GPID-22) comprising 205,371 images across 199 classes and 22 plant species. Unfortunately, cotton disease and pest images are not yet included in this dataset; we are currently working to supplement it. The dataset structure and the architecture of the proposed method are illustrated in Figure 6.

This section reviews the application of computer vision in cotton disease and pest detection, with a focus on image classification and object detection. Studies demonstrated that models such as CNN, VGG16, and DenseNet significantly enhance classification accuracy, while YOLO variants, Faster R-CNN, and Transformer-based methods improve real-time detection performance. Advancements including attention mechanisms, multi-scale feature fusion, knowledge graphs, and edge computing further improved the adaptability of these models for mobile platforms and agricultural machinery. Nonetheless, challenges persist in dataset diversity and generalization, variability in field lighting, and the dynamic expression of disease symptoms. Future research may benefit from integrating time series data and multispectral imaging to further improve detection accuracy and support intelligent pest and disease management in precision agriculture.

#### 2.3.2. Early Warning Systems and Precision Prevention and Control Strategies

In modern agriculture, early warning and precise control of cotton diseases and pests are critical to crop health and yield [82]. Traditional monitoring methods, which rely on manual field inspections, often suffer from delays and high error rates [83]. With advancements in big data, the IoT, DL, and remote sensing technologies, intelligent early warning systems emerged as essential tools for effective disease and pest management.

In recent years, researchers developed early warning systems based on smart sensing, data mining, and artificial intelligence. Cao et al. [84] designed a real-time monitoring system based on agricultural IoT, integrating temperature–humidity, image, and soil moisture sensors. By combining image recognition with data mining, the system achieved a detection accuracy of 95%, representing a 10% improvement over traditional methods. Sitokonstantinou et al. [85] utilized Sentinel-2 satellite data within a semi-supervised learning framework, applying pseudo-labeling to enhance the training dataset and employing random forest regression to predict cotton growth status, enabling precise early warnings. Islam et al. [86] proposed a deep learning and transfer learning-based method for cotton disease detection and developed an intelligent web application that allows farmers to upload leaf images for automatic identification, thereby improving the practicality of pest and disease control.

This section discusses early warning systems for cotton diseases and pests based on big data and intelligent analytics. Research indicates that the integration of IoT, deep learning, and remote sensing significantly improves real-time monitoring and detection accuracy, offering intelligent and cost-effective solutions that surpass traditional methods.

However, current systems mainly emphasize detection and early warning, with limited advancement in integrating precision control strategies. For example, the development of scientifically informed measures—such as pesticide application, biological control, and agronomic practices tailored to pest type, severity, and crop growth stage—remains underexplored. Furthermore, the adoption of intelligent technologies is impeded by limited technical literacy among farmers, skepticism toward new systems, and inadequate network infrastructure in rural regions. Future research should focus on the following:

- Integrating early warning with precise, evidence-based control strategies;
- Designing intuitive, user-friendly system interfaces;
- Adapting technologies to suit resource-constrained agricultural settings to facilitate broader adoption in precision pest and disease management.

## 3. The Application of Deep Learning in Cotton Growth Management

### 3.1. Crop Growth Monitoring and Health Assessment

#### 3.1.1. Cotton Growth Monitoring

Previously, Jia et al. [87] employed traditional image processing and statistical analysis methods to monitor cotton growth and nitrogen status. However, these methods exhibited limited generalization ability, as model parameters were highly sensitive to cotton variety and growth stage, thereby restricting applicability. Zhao et al. [88] proposed a linear regression model based on canopy spectral reflectance indices to predict cotton growth parameters. Nonetheless, the predictive performance was constrained by index saturation and the model’s inability to capture the nonlinear relationships inherent in cotton growth dynamics. In the domain of machine learning, Yang et al. [89] integrated the coefficient of variation method with a random forest model to retrieve cotton growth status. While this approach improved monitoring accuracy, it relied heavily on manually selected features and predefined vegetation indices, limiting its capacity to fully exploit complex spectral information and thereby affecting model adaptability and generalization.

**Figure 7:**
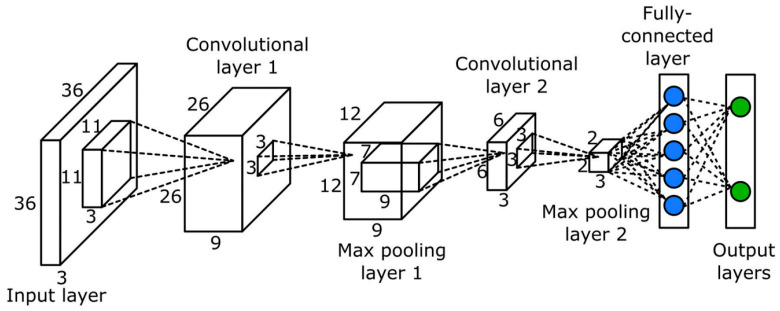
Structure of the convolutional neural network [92].

Deep learning offers a novel solution for cotton growth monitoring by automatically extracting growth status information through efficient algorithms, thereby improving detection accuracy and reducing manual intervention [90]. Feng et al. [91] employed a pre-trained ResNet18 model to estimate the number of cotton seedlings and canopy size from individual image frames, providing more intuitive growth monitoring data for cotton cultivation. Rui Xu, Changying Li, and colleagues [92] utilized convolutional neural networks (CNNs) to detect and count cotton flowers as an indicator of growth status. As shown in Figure 7, their CNN model achieved an accuracy of 0.94 on the training dataset.

In addition, Jiang et al. [93] proposed a deep learning-based method for cotton flowering detection, termed DeepFlower, which provides an efficient tool for characterizing flowering patterns in plants with complex canopy structures such as cotton. Their study demonstrated that the FrRCNN5-cls model trained with five annotation categories achieved a 3% higher average precision for flower detection compared to the FrRCNN3-cls model trained with three categories, offering valuable insights for flowering stage monitoring and cultivar development. Wang et al. [94] designed a lightweight CNN model that utilizes a low-cost monocular camera to identify cotton growth stages, supporting decision-making for precision spraying. Jin et al. [95] introduced an improved MobileViT-based deep learning algorithm that integrates efficient channel attention, depthwise separable convolution, and the MobileOne module, significantly enhancing model performance, particularly in the task of cotton moisture status recognition.

This subsection reviews the application of DL in cotton growth monitoring, emphasizing the potential of computer vision to enhance detection accuracy and efficiency compared to traditional manual methods. Despite significant progress, key challenges remain:

- Lighting variations: while preprocessing can mitigate illumination effects, extreme lighting and complex field environments (e.g., dynamic shadows) still affect detection stability, limiting practical deployment.
- Generalization limitations: models trained on data from specific cotton fields often lack validation across diverse regions, varieties, soils, and climates, reducing their adaptability.
- Model complexity: high computational demands and large parameter sizes hinder deployment on resource-limited platforms such as UAVs and field devices, necessitating lightweight, efficient architectures.

Future developments in deep learning, multimodal data fusion, and smart sensing are expected to advance the intelligence and scalability of cotton growth monitoring, promoting sustainable precision agriculture.

#### 3.1.2. Yield Prediction

Early studies on cotton yield prediction primarily relied on traditional regression models—such as quadratic, pure quadratic, interaction, and polynomial regression—which struggled to capture complex nonlinear relationships and handle high-dimensional data. Zhao et al. [96] leveraged high spatiotemporal resolution imagery from UAVs and sensors, employing a Bayesian Neural Network to predict yield. The model effectively managed irregular data and quantified uncertainty. Similarly, Tugba et al. [97] applied an artificial neural network using limited meteorological and remote sensing inputs to predict yield in data-scarce regions, demonstrating strong performance and practical utility for agricultural decision-making. While effective in specific contexts, traditional machine learning models often exhibit poor generalization in complex environments and require significant computational resources for training and optimization, limiting their scalability in resource-constrained settings [98,99].

Deep learning offers a novel and powerful approach to cotton yield prediction. Unlike traditional methods, deep learning models can automatically integrate multi-source data and extract hierarchical features through convolutional and pooling layers without the need for manual feature engineering [24]. In crop yield forecasting, deep learning demonstrated notable advantages [100]. Xu et al. [101] applied a U-Net-based ENVINet-5 model for pixel-level segmentation of visible and multispectral remote sensing data, followed by a Bayesian regularized neural network for yield prediction. Similarly, Oikonomidis et al. [102] proposed a hybrid deep learning model to investigate the influence of different data features on crop yield prediction. Long et al. [103] employed shared dense layers to extract latent features from multidimensional data and used multi-task subnetworks to predict yields across different years, achieving consistent and low prediction errors both temporally and spatially. Kang et al. [104] introduced a two-step convolutional neural network strategy to progressively downscale the spatial resolution of solar-induced chlorophyll fluorescence products from 0.05° to 0.0005°, demonstrating strong performance in cotton yield estimation tasks.

In addition, Niu et al. [105] utilized UAV-acquired RGB images to generate orthomosaic maps and developed an integrated framework comprising five customized CNN models. Experimental results show that their CNN regression model outperformed conventional CNNs across multiple image scales, achieving an R^2^ greater than 0.9, with a mean absolute error (MAE) of 3.08 pounds and a mean absolute percentage error (MAPE) of 7.76% in row-level yield prediction tasks. This demonstrates the effectiveness of combining UAV imagery with CNN regression models for enhancing precision agriculture. Zhang et al. [106] proposed the YOLO SSPD model based on the YOLOv8 architecture, incorporating spatial-to-depth convolution (SPD-Conv), stride-free convolution, and a simple parameter-free attention mechanism (SimAM), which showed excellent performance in cotton boll detection tasks.

This section reviews cotton yield prediction studies, highlighting the effectiveness of deep learning models under diverse spatial and temporal conditions [107,108]. Integrating UAV imagery with DL enables accurate and efficient pre-harvest yield estimation, offering valuable support for precision agriculture [109]. Nonetheless, key challenges remain:

- Model generalization: Most models are region-specific and lack cross-regional validation. Expanding datasets to cover diverse environments is essential to enhance adaptability and robustness.
- Quality of remote sensing data: UAV and satellite imagery are affected by weather, sensor precision, and acquisition methods, impacting model performance. Improved preprocessing techniques—such as denoising, illumination correction, and enhancement—are needed to ensure data consistency.
- Long-term prediction: Current models often focus on intra-season forecasts, with limited attention to multi-year trends. Incorporating multi-year remote sensing and meteorological data with temporal models (e.g., Transformers, Bi-LSTM) could improve long-term forecasting and strategic planning.

With continued advances in deep learning, multimodal data integration, and intelligent sensing, cotton yield prediction is expected to become increasingly precise and scalable, supporting optimized field management and sustainable agricultural production.

### 3.2. Intelligent Lrrigation and Fertilization

Traditional cotton irrigation methods—such as furrow, sprinkler, and drip irrigation—often suffer from poor uniformity, low water use efficiency, and dependence on manual operation and empirical judgment [110,111,112,113]. To address these limitations, researchers explored the integration of machine learning into intelligent irrigation systems. Phasinam et al. [114] developed an IoT- and cloud-based smart irrigation system using traditional machine learning models (e.g., SVM, random forest, Naïve Bayes) to predict crop water needs. However, these models struggled with time series analysis and complex pattern recognition. Singh et al. [115] employed gradient-boosted regression trees, random forest regression, multiple linear regression, and elastic net regression for soil moisture prediction, improving irrigation precision. Nonetheless, limitations remain due to their reliance on manually crafted features and potential performance bottlenecks as data volume increases.

Deep learning demonstrated strong potential in optimizing water and fertilizer use by enabling precise, data-driven irrigation decisions. These models integrate sensor inputs and real-time climate data to automate irrigation processes and reduce manual intervention [116,117]. Chen et al. [118] proposed a distributed actor–critic deep reinforcement learning model for intelligent irrigation, outperforming traditional methods in decision-making accuracy, resulting in increased cotton yield and improved water use efficiency. Ramirez et al. [119] introduced an AI-driven IoT framework for large-scale agriculture, achieving a 20–35% reduction in irrigation water and a 15–30% reduction in fertilizer usage, without compromising crop yield. Magesh et al. [120] developed a CNN-based image classification system integrated with sensor data for adaptive cultivation, achieving 97.14% accuracy on test data and maintaining over 90% accuracy under non-standard conditions, with prediction errors kept within one classification level.

**Figure 8:**
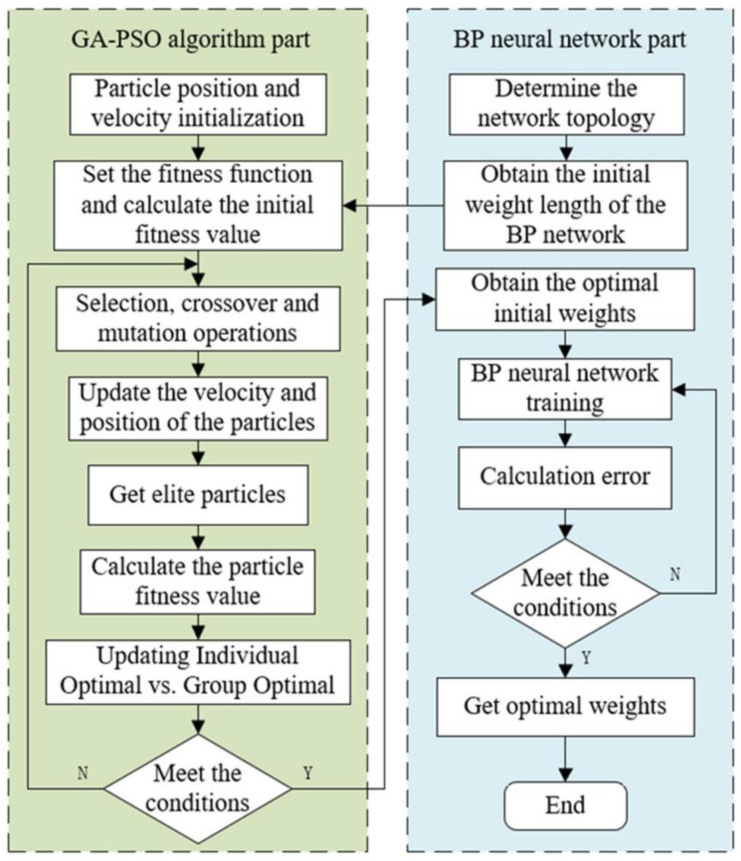
GA–PSO-BP algorithm modeling process [121].

To address fertilizer overuse and the resulting environmental degradation in China’s major crops (cotton, wheat, and tomato), Zhu et al. [121] proposed a BP neural network PID control algorithm optimized via genetic algorithm and particle swarm optimization. As illustrated in Figure 8, experimental results demonstrate that the method significantly improves fertilization precision, reduces resource waste, and mitigates environmental impact. Sami et al. [122] developed a deep learning model based on LSTM networks for accurate prediction of key parameters such as temperature, humidity, and soil moisture, showing strong reliability and predictive performance.

This section reviews the integration of deep learning and IoT in water and fertilizer management, highlighting its pivotal role in intelligent irrigation, yield improvement, and efficient resource allocation in cotton-producing regions [123]. These systems are especially valuable in water-scarce areas, where they help minimize water waste and support sustainable agriculture. However, several challenges remain:

- Model interpretability: deep learning models, though effective in uncertainty management, often lack transparency, limiting their applicability in agricultural settings that demand traceable and explainable decision-making.
- Data transmission and control limitations: in large-scale farms, data transmission delays—exacerbated by poor network infrastructure or high data volumes—can compromise real-time control, underscoring the need for efficient transmission protocols and edge computing.
- Data scarcity: In regions with limited or unstable meteorological and remote sensing data, model performance may degrade. Robust preprocessing and imputation techniques are essential to enhance model generalization and reliability.

Looking ahead, intelligent irrigation systems are expected to play an increasingly vital role in precision agriculture. Future research should focus on the following:

- Integrating explainable AI (XAI) to improve model transparency and usability;
- Advancing edge computing and data transmission frameworks for real-time responsiveness;
- Enhancing data imputation and augmentation to support model performance in data-limited environments.

These advancements will promote the broader adoption of intelligent irrigation systems, fostering more efficient and sustainable water management for cotton and other major crops.

### 3.3. Weed Detection and Precision Weed Control

#### 3.3.1. Field Weed Identification

Cotton’s long growth cycle, wide planting spacing, and slow early development make it highly susceptible to severe weed infestation. Traditional weed control methods, primarily mechanical weeding, often require multiple passes and may be ineffective against certain weed species [124], resulting in low efficiency and potential environmental risks [125]. To address this, Shen et al. [126] proposed an image recognition method based on color features, leveraging the dark red stems of cotton seedlings to distinguish them from green weeds. While effective under controlled conditions, the method relies on manually designed features and image processing steps, making it sensitive to lighting, soil color, and other environmental variations—thus limiting its generalizability in diverse field scenarios.

Compared to traditional methods, deep learning algorithms offer more efficient and accurate weed identification and localization, enabling intelligent weed management in cotton fields. Gallo et al. [65] utilized YOLOv7 for agricultural weed detection, while Jin et al. [127] applied a CenterNet-based approach that detects plant structures to indirectly identify weeds, bypassing the complexity of direct weed classification. Reedha et al. [128] employed a Transformer-based model for UAV imagery, demonstrating that Vision Transformer performs well even with limited annotations. Ajayi et al. [129] trained YOLOv5 on satellite imagery to effectively distinguish weeds from crops. Likewise, Zhao et al. [130] developed the LettWd-YOLOv8l model, integrating global and coordinate attention mechanisms to accurately detect and classify six common weed species. Although not all models have been directly applied to cotton fields, their methodologies are highly transferable, highlighting the strong potential of deep learning in advancing intelligent weed management in cotton production.

**Figure 9:**
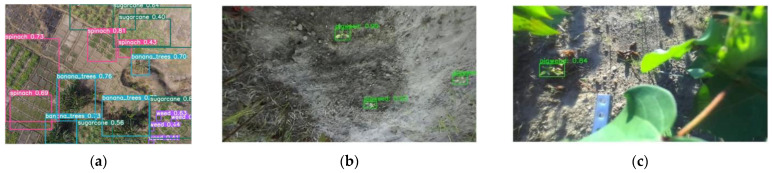
(**a**) Automatic crop type and weed recognition displaying precision values [129]; (**b**) Palmer amaranth weed detection in the cotton field; and (**c**) detecting weeds in presence of shadows during lower sunlight [132].

Moreover, deep learning techniques demonstrated promising results in cotton field weed identification. Rahman et al. [131] addressed the problem of weed detection in cotton fields by constructing an RGB image dataset containing 848 annotated images featuring three common weed species. They evaluated 13 object detection models—including YOLOv5, RetinaNet, EfficientDet, and Faster R-CNN—for weed identification, providing technical support for precision weed management in cotton cultivation. Mwitta et al. [132] successfully applied the YOLOv4-tiny model to detect Palmer amaranth weeds in cotton fields, achieving a weed removal rate of 72.35%. These findings further demonstrate the feasibility of deep learning in supporting effective weed control in cotton field environments. Figure 9 displays some examples of deep learning-based weeds detection.

This section reviews the application of deep learning in weed detection and management in cotton fields, emphasizing the advantages of machine vision and object detection algorithms in enhancing detection accuracy and automation. Studies have shown that integrating deep learning with machine vision enables precise identification and targeted treatment of individual weed species [133], thereby minimizing yield loss and quality degradation caused by weed infestation. However, practical challenges remain:

- Complex field environments and weed variability: dynamic conditions—such as fluctuating lighting, soil backgrounds, and weed growth stages—can lead to recognition errors and reduce detection robustness.
- Limited generalization: many models are trained on region-specific datasets and recognize only a few weed species, with uncertain performance on unseen types or under diverse conditions.
- Short-term focus: Most research emphasizes single-season detection, with limited assessment of long-term weed control impacts on cotton growth and yield.

In the future, with continued advancements in deep learning, multimodal data integration, and agricultural robotics for weeding, cotton field weed detection and precision management are expected to become increasingly intelligent. To address the aforementioned challenges, future research should focus on the following directions:

- Expanding dataset diversity to enhance model generalization across different environments, lighting, soil types, and weed stages;
- Incorporating multispectral and hyperspectral imaging to improve species-level discrimination, especially when weeds and crops have similar visual features;
- Developing long-term monitoring frameworks using temporal models (e.g., LSTM, Transformer) to assess the prolonged effects of weed management strategies on crop performance.

Through these improvements, deep learning-based weed detection technologies in cotton fields are expected to achieve more efficient and accurate intelligent weed control, thereby providing stronger technical support for precision agriculture.

#### 3.3.2. Weeding System and Intelligent Spraying System

A. Weeding System

Despite technological advancements, cotton growers still face major challenges such as herbicide-resistant weeds—particularly glyphosate-resistant species—and labor shortages. Traditional weeding methods are increasingly inadequate for the precision demands of modern agriculture [134]. Manual labor long dominated cotton field management, but labor-intensive practices are no longer sustainable. Although machinery such as tractors can aid weed removal, they risk damaging cotton plants. Chemical herbicides, while effective in the short term, led to rising weed resistance and environmental concerns, limiting their long-term viability [135].

In recent years, the integration of deep learning and agricultural robotics significantly advanced the development of intelligent mechanical weeding robots. As eco-friendly alternatives to chemical herbicides, these robots offer advantages in cost-effectiveness and environmental sustainability, making them a key focus in agricultural automation research [136,137]. Quan et al. [138] developed an inter-row weeding robot using YOLOv3 to detect crops and weeds, establishing protective zones to minimize crop damage and define precise weeding areas. Chang et al. [139] proposed a smart weeding system based on deep convolutional neural networks, featuring a modular, inverted pyramid-shaped weeding tool mounted on a mobile platform. This design achieved high precision and effective weed coverage without soil contamination. Ilangovan et al. [140] introduced the Weedbot system, which integrates CNN and Faster R-CNN for weed classification in rice fields, followed by robotic arm-based weed removal. Notably, our recent research proposed a YOLOv11l-based intra-row weeding robot for lettuce [141]. While it has not yet been applied to cotton fields, the system exhibits strong potential for adaptation to cotton weeding scenarios.

Although most existing studies focus on crops other than cotton, deep learning-based robotic weeding systems show strong potential for application in cotton fields. Cotton Incorporated, in collaboration with multiple research institutions [142], developed a weed recognition system based on the Husky robotic platform, integrating YOLOv3 and the CLoDSA data augmentation tool. This system autonomously identifies weeds and cotton plants, plans operation paths, and avoids cotton seedlings, achieving an average recognition accuracy of 89% and a weed removal efficiency exceeding 85%. Similarly, Fan et al. [143] applied an improved Faster R-CNN model for precise weed detection during the cotton seedling stage and integrated it into an autonomous spraying robot, enabling precision-targeted weed control. These studies highlight the growing potential of deep learning and intelligent robotics to revolutionize weed management in cotton cultivation.

This section reviewed the application of deep learning-enabled intelligent weeding robots in cotton field management. Studies have shown that AI-powered weeding robots can accurately identify and predict weeds, enabling targeted interventions and enhancing the efficiency and sustainability of weed control [144]. Deep learning also improves the adaptability of these robots to complex field conditions and dynamic environments [18], providing new directions for future weeding technologies. Despite these advances, several challenges remain:

- Limited generalizability and modularity: Most current systems are crop-specific and lack versatility. Future research can focus on modular tool designs that support quick attachment changes or develop multi-crop-compatible intelligent weeding platforms.
- Navigation and energy limitations: Autonomous navigation and energy supply remain critical bottlenecks. Enhancing localization accuracy and reducing reliance on human intervention, along with integrating renewable energy sources such as solar power, could improve performance and sustainability in large-scale operations.

Looking ahead, continued progress in deep learning, agricultural robotics, precision navigation, and energy management will position intelligent weeding robots as key tools for improving cotton field management, reducing environmental impact, and lowering production costs—ultimately advancing the automation of sustainable agriculture.

B. Intelligent Spraying System

Traditional chemical weeding methods often rely on manual operations or large-scale machinery, posing health risks to operators and lacking precision. This frequently results in herbicide overuse, contributing to environmental degradation and the emergence of resistant weed species [143]. Additionally, herbicide drift and inefficient application can lead to soil and water contamination, threatening the sustainability of agricultural ecosystems [145]. To improve spraying precision, Chen et al. [146] developed a defoliant application decision model combining a backpropagation neural network with Bayesian regularization and UAV remote sensing imagery. While the model improved spraying accuracy, its limited complexity hindered its ability to capture nonlinear patterns and high-dimensional features. Moreover, issues such as insufficient or imbalanced data may lead to overfitting, reducing the model’s generalization capability.

In recent years, deep learning has driven major advancements in intelligent spraying systems. By integrating deep learning with smart mechanical platforms, these systems can accurately detect weed-infested or diseased areas and apply chemicals selectively, thereby reducing pesticide usage, minimizing environmental pollution, and improving spraying efficiency and safety. Latif et al. [147] developed a UAV-based cognitive vision system using ResNet for automated plant disease recognition and precision spraying. Trained on 70,295 leaf images across 38 disease classes, the system achieved 99.78% accuracy, offering a cost-effective solution for precision agriculture. Li et al. [148] designed a real-time spraying system based on an improved YOLOv5 model with a MobileNetv3 backbone and a squeeze-and-excitation attention mechanism, achieving a pesticide hit rate of 90.80% and significantly improving chemical application efficiency. Sabóia et al. [149] combined Faster R-CNN and YOLOv3 in a real-time selective spraying system targeting *Ipomoea* weeds in cotton fields, achieving an 81% weed control rate and reducing pesticide use in non-target areas.

Current research indicates that deep learning-enabled intelligent spraying systems offer substantial potential for precise control of pests, diseases, and weeds, improving spraying efficiency while mitigating the environmental impact of chemical applications. However, several challenges remain:

- Coordinated management in large-scale operations: In expansive fields, multiple UAVs or autonomous sprayers require effective task allocation, communication, and collaborative control. Optimizing path planning, avoiding redundant spraying, and coordinating task distribution are key research priorities;
- Precision spraying and environmental protection: While systems can adjust pesticide type and dosage based on disease classification, further improvements are needed to ensure chemicals are applied exclusively to target vegetation. Integration of path optimization, spray control algorithms, and advanced nozzle technologies is essential;
- Robustness under complex lighting conditions: Reduced recognition accuracy under shaded or uneven lighting remains a concern. Solutions may include multispectral imaging, high dynamic range techniques, and adaptive illumination correction algorithms.

Future advancements in deep learning, robotics, sensor technology, and precision equipment are expected to further enhance intelligent spraying systems. Key research directions include the following:

- Developing collaborative control strategies for multi-device coordination;
- Enhancing environmental adaptability to maintain accuracy in complex conditions such as dense vegetation and low-light environments;
- Integrating multi-sensor fusion (e.g., RGB, thermal, and multispectral) to improve decision reliability in precision spraying.

With these innovations, intelligent spraying systems are poised to become a cornerstone of pest and weed control in precision agriculture, driving a more intelligent, efficient, and sustainable transformation of cotton production.

## 4. Application of Deep Learning in Cotton Harvesting and Processing

### 4.1. Intelligent Harvesting Robot

Cotton-picking robots are a key innovation in modern agriculture. By integrating computer vision, deep learning, and reinforcement learning, these systems can autonomously perform harvesting tasks, greatly enhancing efficiency and reducing labor costs. For instance, Gharakhani [150] developed a multi-finger end-effector cotton-picking robot, while Thapa et al. [151] proposed an improved multi-boll harvesting system—both demonstrating the potential of intelligent harvesting technologies. Unlike traditional methods that rely on manual labor or basic mechanical tools, intelligent robots can accurately identify the location, maturity, and quality of cotton bolls in real time, enabling fully automated harvesting through computer vision-driven decision-making.

**Figure 10:**
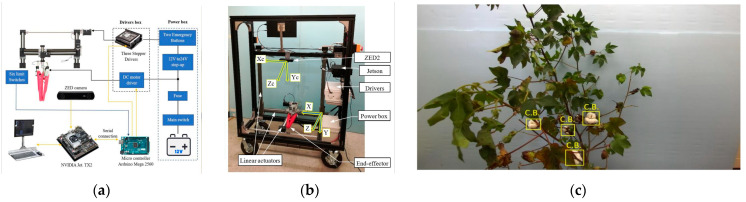
(**a**) Functional block diagram. (**b**) Cotton harvesting robot system setup. (**c**) A sample inference image of YOLOv4-tiny on cotton bolls (C.B.). Reprinted with permission from Ref. [150]. Copyright 2023, Hussein Gharakhani.

Computer vision is a core technology underpinning intelligent cotton-picking robots. Equipped with high-resolution cameras and deep learning models, these robots can acquire real-time field imagery, accurately identify cotton boll features, and perform target localization and motion planning [152]. For instance, the multi-finger end-effector robot developed by Gharakhani et al. [150] integrates a stereo camera with the YOLOv4-tiny algorithm, achieving a 72% picking rate and an average cycle time of 8.8 s per boll. Finally, they deployed the deep learning models on their self-developed robot and tested the detection, localization, and harvesting system in the cotton fields at the R. R. Foil Plant Science Research Center, Mississippi State University’s Agricultural and Forestry Experiment Station. As illustrated in Figure 10, the system’s workflow, mechanical design, and detection performance underscore the value of deep learning in enabling precision cotton harvesting.

Computer vision also plays a vital role in robot navigation, enabling efficient movement within cotton fields. Oliveira et al. [153] developed a wheeled autonomous robot equipped with multiple cameras to capture ground and crop row information. The system uses visual feedback to adjust movement direction, ensuring stable row-following navigation. Meanwhile, they deployed the model on the robot and conducted experimental validation in real cotton farms. Mwitta et al. [132,154] employed a fully convolutional network for path detection, achieving 93.5% pixel accuracy. By integrating GPS and a dynamic window approach for path planning, their robot autonomously navigates while avoiding obstacles, reducing the risk of crop damage and improving the stability of the harvesting process. They placed the robot equipped with the research algorithm in the cotton test fields at the University of Tennessee, conducting extensive experiments in real cotton fields to verify the feasibility and effectiveness of the model in practical scenarios.

After computer vision provides positional data, effective path planning is essential to enhance harvesting efficiency. Reinforcement learning (RL), a key branch of deep learning, has shown strong potential in optimizing robot paths in complex agricultural environments. Wang et al. [155] proposed an improved deep Q-network that uses robot position and target coordinates as inputs, employing an ε-greedy strategy to enhance path search efficiency. Yang et al. [156] introduced a Residual-like soft actor–critic model, combining a residual structure with the soft actor–critic algorithm for robust path planning and navigation decisions. Wang et al. [157] developed a deep RL-based coverage path planning method for a kiwi-picking robot, achieving rapid convergence, reduced path redundancy, and improved operational efficiency. Although RL applications in cotton harvesting are still limited, successful implementations in orchard environments demonstrate its potential to help robots adapt to variable field conditions—such as changing climate, soil types, and crop row structures—making it highly promising for future cotton harvesting systems.

This section reviews the application of computer vision, deep learning, and RL in cotton harvesting robots, demonstrating their ability to accurately identify cotton bolls, plan optimal harvesting paths, and autonomously avoid obstacles—thereby enhancing the automation level of cotton harvesting. However, several challenges remain [158]:

- Reliance on prior knowledge for obstacle avoidance: Most current algorithms depend on predefined information, limiting their ability to respond to unknown obstacles. Future research should explore self-supervised learning and model-free reinforcement learning to enable autonomous learning in complex environments.
- Limited navigation performance in complex terrains: Navigation accuracy and stability remain insufficient in scenarios involving curved paths, uneven terrain, and crop row transitions. Integrating multimodal sensor fusion (e.g., RGB-D cameras and LiDAR) and fusion technologies [159] may improve adaptability.
- Lack of open agricultural datasets: The scarcity of standardized datasets constrains model training and evaluation. Establishing large-scale, publicly available agricultural image and path datasets would enhance the generalization of deep learning models and accelerate intelligent equipment development.

Looking ahead, advancements in computer vision, deep reinforcement learning, and sensor fusion will drive cotton harvesting robots toward greater precision, efficiency, and autonomy. Key research directions include the following:

- Developing adaptive obstacle avoidance strategies through autonomous learning in unknown environments;
- Optimizing path planning by combining reinforcement learning with dynamic search algorithms to improve performance in complex field conditions;
- Building comprehensive agricultural datasets to support robust model training and improve real-world applicability.

In sum, intelligent cotton harvesting robots, powered by advanced AI and robotics technologies, hold great promise for reducing labor dependence, increasing harvesting efficiency, and promoting the sustainable development of precision agriculture.

### 4.2. Cotton Quality Inspection and Grading

#### 4.2.1. Fiber Quality Inspection

Cotton fiber quality directly influences both market value and textile performance. Traditional assessment methods rely heavily on manual inspection, which is labor-intensive, prone to human error, and inefficient for large-scale sample analysis. With the advancement of deep learning, fiber quality detection has become increasingly automated and intelligent, enabling more accurate, efficient, and scalable evaluation processes.

**Figure 11:**
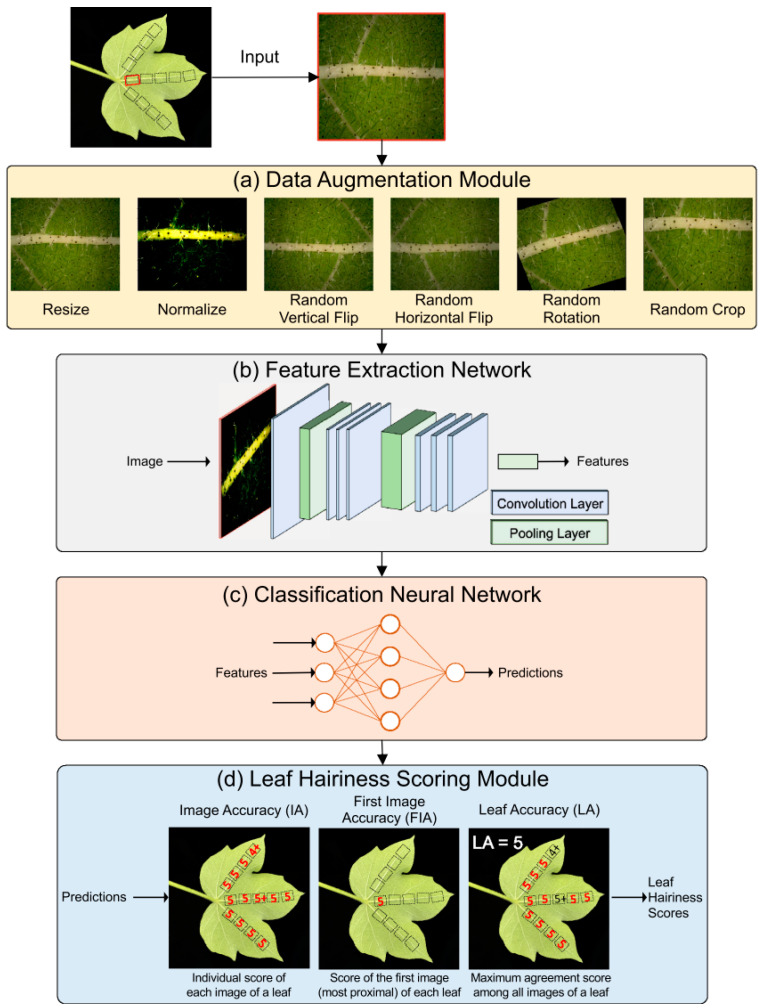
Network architecture of the proposed deep learning model to score cotton leaf hairiness [160].

Deep learning advanced fiber quality detection by automating feature extraction, classification, and evaluation processes. For instance, Rolland et al. [160] developed HairNet (Figure 11), comprising four modules: (a) data augmentation, (b) feature extraction, (c) classification, and (d) leaf hairiness scoring. The network achieved 89% image-level and 95% leaf-level classification accuracy for cotton leaf pubescence across the dataset. Dai et al. [161] proposed a GRU model integrated with an attention mechanism to automatically identify key features influencing yarn quality, thereby enhancing prediction accuracy. This method outperforms traditional BP neural networks and addresses the lower temporal modeling capability of standard LSTM models when handling time series data.

Additionally, Geng et al. [162] proposed a non-destructive cottonseed fiber content detection method based on MobileNetV2 and transfer learning, achieving an average classification accuracy of 98.43%, significantly enhancing both efficiency and accuracy. Wang et al. [163] developed a method for detecting and classifying foreign fibers in cotton using polarization imaging combined with an improved YOLOv5 algorithm. The enhanced model exhibited strong robustness under varying lighting conditions, fiber types, positions, and sizes, demonstrating high resistance to environmental interference.

Through the discussion in this section, deep learning models demonstrated the ability to automatically analyze large volumes of cotton samples and extract valuable quality information, significantly enhancing the automation of fiber quality detection. As deep learning technology advances, CNN-based quality detection methods are expected to continue evolving, driving the cotton industry towards greater efficiency and precision. However, several challenges remain:

- The quality of image capture may be influenced by lighting conditions and the positioning of samples, necessitating further optimization of hardware design to minimize the impact of environmental factors.
- For most models, detecting small targets remains a challenge. For instance, the detection capability of the model may decrease when identifying foreign fibers smaller than 0.5 mm^2^.

#### 4.2.2. Cotton Impurity Identification

Cotton color and impurity recognition are critical factors influencing fiber quality and market value. Traditional detection methods rely on manual visual inspection, which is inefficient, prone to subjective bias [164], and typically constrained to controlled laboratory environments, limiting their applicability for large-scale field detection [165]. Some studies explored image color-based impurity detection—for example, Tantaswad et al. [164] implemented a color-based method, but its performance is highly sensitive to ambient lighting and lacks generalization. Machine learning approaches have also been applied; Fisher et al. [166] used a random forest algorithm for cotton grading, though its scalability to large datasets is limited. In contrast, deep learning models achieved up to 98.9% accuracy in grading Chinese upland cotton, demonstrating strong potential for automated, scalable, and high-precision cotton quality evaluation.

Deep learning-based cotton color classification and impurity recognition technologies significantly improve sorting efficiency while reducing human error. Zhang et al. [167] applied YOLOv4 for impurity detection in machine-harvested cotton, incorporating CIOU loss and an improved focal loss to optimize performance. On a dataset of 100 images, the method achieved an average recognition rate of 94.1%, demonstrating its practical value in impurity detection. Li et al. [168] developed Cotton-Net, a model for rapid impurity content detection in mechanically harvested seed cotton. Additionally, Xu et al. [169] proposed a lightweight model based on improved YOLOv4-tiny, achieving detection accuracies of 98.78% for white impurities and 98.00% for cotton-colored impurities. Similarly, Jiang et al. [170] enhanced YOLOv8 and introduced Cotton-YOLO-Seg, which significantly improved impurity rate detection and enabled accurate segmentation of cotton fibers from impurities, further advancing the automation of cotton quality grading. Additionally, as edge-based cotton grading systems grow in scale, ensuring fault tolerance through strongly connected topologies such as the bubble-sort star graph or the locally twisted cube can be vital [171,172]. These structures guarantee system functionality even under partial node or link failures.

The impurity content in mechanically harvested seed cotton is typically high and unevenly distributed, making impurity rate a key indicator for assessing harvest quality and grading [170]. Deep learning technologies demonstrated strong potential in accurately detecting cotton impurities and enabling rapid on-site assessment, significantly contributing to cotton quality management. However, several challenges remain in practical applications:

- Limited datasets and poor model generalization: Most existing datasets are derived from controlled environments and fail to represent the variability of real-world conditions, such as lighting changes, background interference, and regional cotton color differences. Enhanced testing and optimization in diverse field and factory settings are needed to improve model robustness and generalizability.
- High computational complexity limiting real-time performance: On high-speed production lines, some deep learning models are too computationally intensive, hindering real-time detection. Future research should prioritize lightweight architectures (e.g., MobileNet, ShuffleNet) and employ techniques such as model pruning, quantization, and edge computing to enhance deployment efficiency.

In the future, advancements in deep learning, computer vision, and edge computing will further improve the precision, speed, and intelligence of cotton impurity detection and grading systems. Key research directions include the following:

- Expanding dataset size to enhance model generalization, ensuring high accuracy across varying lighting conditions, backgrounds, and cotton varieties;
- Optimizing model structures using lightweight CNNs, Transformers, and adaptive enhancement methods to ensure real-time, efficient detection;
- Incorporating multi-modal sensing technologies (e.g., polarization imaging, NIR spectroscopy) to enhance impurity recognition under complex conditions.

In sum, deep learning-based cotton color classification and impurity recognition hold great promise for advancing automated cotton quality detection. With larger datasets, more efficient models, and improved hardware integration, this technology will play a pivotal role in promoting precision, efficiency, and intelligence in the cotton industry.

## 5. Discussion

### 5.1. Challenges

#### 5.1.1. High Cost of Data Acquisition and Annotation

Deep learning models offer powerful capabilities for modeling complex nonlinear relationships in cotton cultivation, enabling high-accuracy predictions that are critical for understanding intricate agricultural interactions. However, the development of high-quality models depends heavily on large volumes of labeled data, and the associated costs of data collection, annotation, and processing remain substantial. Bishshash et al. [173] conducted field data collection at the Cotton Research Institute using multiple devices, along with tripods, light shields, and other auxiliary equipment to ensure image quality. Additionally, professional annotators were hired for labeling, further increasing research costs. This underscores the significant challenges of data acquisition and labeling, particularly in the early stages of deep learning model development.

To reduce the high cost of data acquisition, some research teams publicly released high-quality agricultural datasets, providing a foundation for the application of deep learning and computer vision in agriculture. For example, Bishshash et al. [173] constructed a cotton leaf disease dataset containing 2137 raw images and trained an Inception V3 model, achieving 96.03% accuracy on the validation set—demonstrating the effectiveness of deep learning in plant disease detection. Muzaddid et al. [174] developed TexCot22, the first publicly available cotton boll tracking dataset, comprising approximately 150,000 labeled instances, with an average of 70 individual bolls per video sequence. This dataset significantly advanced research on cotton boll tracking and counting systems.

To address the challenge of limited labeled data, researchers explored model optimization and data augmentation techniques to alleviate data scarcity. Amani et al. [175] applied the synthetic minority over-sampling technique to effectively address class imbalance by generating synthetic samples between minority class instances. This approach enhances the model’s learning capability on imbalanced datasets and holds significant potential for tasks such as cotton disease classification and cotton boll detection.

#### 5.1.2. Interpretability Issues of Deep Learning Models

As discussed, deep learning technologies demonstrated high accuracy in tasks such as cotton recognition and prediction. However, model interpretability remains a significant concern. Zhang et al. [176] emphasized that despite the success of deep neural networks across various domains, their inherent “black-box” nature raises questions about transparency. Wu et al. [177] further noted that the highly distributed representations within deep models complicate parameter analysis and regularization, limiting interpretability. Improving model interpretability is essential not only for understanding model behavior, but also for optimizing performance and enhancing the credibility of scientific applications in agriculture. To enhance the interpretability of deep learning models, Dong et al. [178] incorporated semantic information into the training process, encouraging the model to focus on meaningful features while maintaining computational efficiency.

Algorithm unrolling is another promising approach. Monga et al. [179] transformed traditional iterative algorithms into deep network architectures, where each layer corresponds to an iteration step. This method retains the interpretability of the original algorithm while leveraging training data to enhance learning performance. Additionally, Askr et al. [180] combined deep learning with grey wolf optimization and XAI techniques to optimize a ResNet50 model for cotton disease classification. Their model achieved 99% accuracy and provided visual explanations of decision-making processes, significantly improving interpretability and transparency in agricultural applications.

#### 5.1.3. Computational Resource Constraints and Challenges in Practical Deployment

While deep learning achieved remarkable success across various domains, it remains highly resource-intensive, with training and inference requiring significant computational power. In real-world applications, challenges such as limited hardware capacity, high model complexity, and data transmission constraints pose significant obstacles to deployment [181]. Even with sufficient computing infrastructure, the large number of parameters and model file sizes—combined with the complexity of deployment environments and hardware limitations—can result in slow inference speeds and reduced performance on resource-constrained devices. These limitations hinder the practical application of deep learning models in real-time and large-scale agricultural scenarios. Recent developments in fault-tolerant interconnection network topologies, such as bubble-sort star graphs, locally twisted cubes, and enhanced hypercubes, provide promising solutions to improve the robustness of large-scale intelligent systems. These topologies, studied under various diagnosability models, including PMC, MM*, and the novel MC model [171,182,183], offer theoretical frameworks for designing resilient computing architectures. Their potential integration with edge AI applications in agriculture could greatly enhance the deployment reliability of deep learning-based cotton monitoring and processing systems.

### 5.2. Future Perspectives

Deep learning demonstrated substantial potential in cotton cultivation, particularly in plant recognition, disease detection, yield prediction, and quality assessment. However, key challenges, such as high data acquisition costs, limited interpretability, and computational constraints, still hinder its practical deployment. Future research is expected to improve model efficiency, accuracy, and field applicability.

Enhancing interpretability and transparency is a crucial focus area. Despite the success of deep learning in agriculture, its “black-box” nature limits trust and widespread adoption. Future efforts may include:

- Developing XAI techniques, such as attention visualization and causal inference-based explanations, to reveal neural network decision processes;
- Integrating symbolic AI and graph neural networks (GNNs) [20,184,185,186,187] to improve reasoning capabilities and enhance model controllability in complex agricultural scenarios;
- Creating lightweight, transparent models to reduce computational complexity, improve interpretability, and ensure their broad applicability in agriculture, thereby increasing trust in intelligent agricultural systems among farmers and researchers.

Optimizing deep learning models for efficient deployment is essential to improving their practicality in agriculture by reducing computational costs and enhancing real-time performance. Key research directions include:

- Model compression and acceleration: Techniques such as pruning, quantization, knowledge distillation, and low-rank decomposition can significantly reduce model size and computational load. For instance, pruning eliminates redundant weights, while quantization replaces floating-point operations with low-bit integer calculations to accelerate inference [188];
- Lightweight network design: architectures such as MobileNet, ShuffleNet, EfficientDet, and YOLOv7 reduce resource consumption, making them suitable for deployment on mobile and embedded devices [189];
- Advanced architecture exploration: investigating state-of-the-art models such as YOLOv12 can enhance both detection accuracy and processing speed, improving adaptability in dynamic agricultural environments;
- Semi-supervised and self-supervised learning: These approaches reduce dependence on large labeled datasets and improve model generalization across diverse conditions. Additionally, methods such as wavelet interpolation transformations can be employed to further boost model robustness and performance [190,191].

Developing computational infrastructure and edge AI: Deep learning models are typically resource-intensive, limiting their deployment in field conditions. Future efforts should focus on the following:

- Optimizing in-memory computing architectures to reduce data transfer overhead, improve energy efficiency, and support heterogeneous hardware (e.g., GPUs, CPUs, and FPGAs) through universal scheduling frameworks [192];
- Leveraging cloud and distributed computing to provide more powerful training and inference capabilities for deep learning, while integrating cloud–edge collaborative computing to reduce data transmission and enhance the intelligence of agricultural equipment [193];
- Adopting hardware acceleration technologies, such as TensorRT, Google Edge TPU, and FPGAs, to boost model performance and reduce energy consumption [194];
- Designing energy-efficient AI devices to support low-power deep learning applications suitable for agricultural scenarios.

Expanding and optimizing agricultural datasets: High-quality, diverse datasets are foundational for deep learning performance in agriculture. Future directions include the following:

- Constructing multi-view, high-precision datasets that span growth stages, lighting conditions, and regions to improve model generalization;
- Applying transfer learning and few-shot learning to reduce dependence on extensive labeled data, enabling broader applicability in data-scarce regions [195];
- Fusing multi-modal data, such as remote sensing, meteorological, soil moisture, and growth data, to build comprehensive agricultural monitoring systems;
- Promoting open data sharing and establishing standardized cotton datasets to facilitate global collaboration and innovation.

Advancing precision agriculture through deep learning: Deep learning will continue to empower precision agriculture, improving management efficiency and sustainability. Key applications include:

- Early disease detection systems using multi-source data to monitor and control pests and diseases such as fusarium wilt and cotton bollworm;
- Individual plant-level management through computer vision and UAVs for optimizing irrigation, fertilization, and pest control;
- Intelligent spraying and selective weeding systems using object detection to precisely apply agrochemicals, reducing environmental impact and improving efficiency.

Additionally, intelligent cotton supply chains increasingly rely on interconnected sensing and computing nodes. Applying fault-tolerant concepts, such as nature diagnosability [196,197] and matching preclusion [198], may help ensure uninterrupted operation of these systems under hardware or communication failures. Deep learning has shown significant promise in advancing cotton cultivation through improved plant recognition, yield prediction, pest management, and quality assessment. Nevertheless, challenges persist—including high computational demands, expensive data labeling, and limited model interpretability. To realize the full potential of deep learning in agriculture, future research should prioritize model optimization, hardware infrastructure development, dataset expansion, and practical precision agriculture applications. These efforts will drive the digital transformation of agriculture, supporting the cotton industry’s evolution toward greater efficiency, precision, and sustainability.

Data governance and privacy protection: With the application of AI and deep learning technologies in agriculture, data security and privacy protection have become critical issues. Future research should focus on the following aspects:

- The large volumes of data generated during cotton cultivation and processing involve personal information of farmers and agricultural workers. Therefore, how to appropriately handle and protect this data, ensuring that its use complies with ethical and legal standards, is crucial for future development.
- Data sharing and collaboration should follow a governance framework that protects the interests of all parties involved, while also promoting data flow and sharing to support more precise agricultural decision-making.

Inclusivity of AI in agriculture: The widespread adoption of AI technologies may lead to technological inequality, particularly impacting small farms and developing countries. Future developments should consider the following points:

- In regions with limited technological and financial resources, there may be challenges in adopting and benefiting from these technologies, which could further exacerbate the digital divide between rural and urban areas, as well as between countries.
- Future AI applications in agriculture should take into account accessibility across different regions and groups, ensuring that the use of AI does not widen the wealth gap, but instead helps a broader range of farmers and agricultural workers.

Socioeconomic impact: The widespread use of AI technologies will have profound effects on the employment and labor markets for agricultural workers. Attention should be given to the following issues:

- As automation and intelligent equipment become more common, some traditional agricultural jobs may decrease or disappear. Therefore, future research should explore labor retraining and skill transition programs to help affected farmers and workers adapt to the changes brought by new technologies.
- Furthermore, the shift in agricultural production methods due to AI applications may affect agricultural policies, market supply and demand relationships, and global trade dynamics. Therefore, future studies should not only focus on technological advancements, but also conduct in-depth analysis of the potential socioeconomic consequences.

## 6. Conclusions

As a vital economic crop in agriculture and the textile industry, the intelligent transformation of cotton cultivation is essential for ensuring food security, enhancing industrial competitiveness, and promoting sustainable development. While traditional agricultural practices, spectral imaging, and machine learning contributed to advancements in cotton production and supply chain management, they face limitations in addressing complex environments and dynamic production needs. In recent years, deep learning demonstrated powerful capabilities in data analysis and pattern recognition, leading to breakthrough applications in seed optimization, pest and disease detection, intelligent irrigation, robotic harvesting, and fiber grading. Research indicates that deep learning significantly enhances cotton pest control, growth monitoring, yield prediction, smart equipment development, and fiber quality assessment. For instance, hyperspectral imaging combined with deep learning enables accurate seed quality evaluation; smart irrigation systems can dynamically optimize water and fertilizer use, reducing resource waste by 20–35%; and computer vision-based early warning systems and RL-driven path planning are propelling agricultural machinery toward autonomous decision-making, reducing labor costs by over 30% and boosting productivity. Furthermore, deep learning, enabled precision spraying, and genomic data analysis contribute to reduced chemical pollution, improved stress resistance, and environmentally friendly cultivation practices, accelerating the cotton industry’s transition toward sustainable development.

Despite its promising applications, the adoption of deep learning in the cotton industry still faces several challenges, including limited model generalization across regions, low adaptability to dynamic field environments, high computational demands, and the substantial cost of data labeling. Future research should prioritize dataset expansion and standardization, the development of lightweight models, and real-time performance optimization to enhance the stability and scalability of deep learning in complex agricultural settings. Moreover, the integration of multi-modal data—such as remote sensing imagery, meteorological information, and soil moisture content—will be crucial for improving intelligent agricultural decision-making and bridging the gap between laboratory research and field deployment. Through these advancements, deep learning will play an increasingly vital role in the intelligent transformation of the cotton industry, driving improvements in productivity, resource efficiency, and environmental sustainability—ultimately accelerating the global shift toward precision, automated, and sustainable agriculture.

## Figures and Tables

**Table 1:** Literature Selection Criteria.

Criteria Type	Description
Inclusion	1. The research focuses on specific stages within the cotton value chain, such as planting, pest and disease identification, harvesting, grading, and processing.
	2. At least one mainstream deep learning algorithm (e.g., CNN, RNN, Transformer, YOLO) is employed in the study.
	3. The selected papers are published in either Chinese or English to ensure readability and accurate comprehension.
	4. The studies are based on real-world field or factory data, or are validated, rather than purely theoretical models or simulation tests.
	5. All selected papers are peer-reviewed journal or conference papers, ensuring academic rigor and verifiability.
Exclusion	1. The study exclusively uses traditional machine learning methods (e.g., SVM, RF, and KNN), without employing deep learning techniques.
	2. The paper is limited to a technical review or patent literature, without original experimental data or performance results.
	3. The paper lacks a complete model architecture description or experimental validation, such as those that describe methods without performance evaluation.

**Table 2:** Table of task categories and evaluation metrics.

Application Type	Common Models	Common Evaluation Metrics	Typical Tasks/Examples
Image classification	CNN, ResNet, VGG, DenseNet	Accuracy, precision, recall, F1-score, AUC	Cotton species classification, disease type identification, and damage detection
Object detection	YOLO(v5/v8), Faster R-CNN YOLOX, CenterNet	mAP(@50 or @0.5:0.95) precision, recall, FPS	Cotton damage detection, cotton bollworm identification, and disease localization
Image segmentation	U-Net, SegNet, DeepLabV3+	IoU, dice coefficient	Cotton field area segmentation, cotton plant recognition, and cotton leaf lesion area extraction
Regression prediction	LSTM, GRU, MLP, 1D-CNN, Transformer	RMSE, MAE, R^2^, MSE	Cotton yield prediction, vitality forecasting, and growth stage estimation
Multimodal/fusion recognition	CNN + GLCM, Transformer + KGE YOLO + SRNet	Accuracy, mAP, FPS, recall	Hyperspectral + texture feature fusion, knowledge graph-assisted recognition, and small object enhancement detection

## Data Availability

Data will be made available on request.
